# Effects of febuxostat in different doses on uric acid, inflammatory factors of serum and knee articular cavity, endothelin-1, and oxidative stress in patients with a gout-a comparative study

**DOI:** 10.4314/ahs.v24i4.40

**Published:** 2024-12

**Authors:** Ning Tie, Lijie Bai, Hongbin Li, Dafu Man, Lei Wang, Xinlu Zhou, Yong Wang

**Affiliations:** 1 Department of Rheumatology, The Affiliated Hospital of Inner Mongolia Medical University, 010050, China; 2 Clinical undergraduate class of 2015, Inner Mongolia Medical University, Inner Mongolia, 010050, China

**Keywords:** Febuxostat, hyperuricemia, gout, blood uric acid, serum inflammatory factors, inflammatory factors of knee articular cavity, endothelin 1, oxidative stress response

## Abstract

**Objective:**

The present comparative study aimed to investigate the effects of different doses of febuxostat on uric acid, inflammatory factors of serum, knee articular cavity, endothelin-1, and oxidative stress in patients with gout.

**Methods:**

80 cases with hyperuricemia admitted to our hospital (January 2018- March 2020) were randomly distributed into two groups. The control group was administered 40 mg of febuxostat tablets daily., whereas the treatment group was administered febuxostat tablets 80 mg daily. Data were collected from two groups of patients, including uric acid level, TNF-α levels of serum and knee articular cavity, vascular endothelial function, and complications 1 month after the intervention.

**Results:**

After data intervention, the levels of uric acid, TNF-α levels of serum, and knee articular cavity, NO, and SOD were significantly different between the treatment group and the control group (each p< 0.05). There were no significant differences in abdominal pain and diarrhea, liver damage, kidney damage, acute gout, and pruritus between the two groups (p >0.05). The duration of activity disorder, pain duration, and swelling in the treatment group were significantly shorter than those in the control group (p< 0.05). The uric acid level was positively correlated with serum TNF-α level (p < 0.05), and negatively correlated with NO and SOD levels (p < 0.05).

**Conclusion:**

For hyperuricemia-induced gout patients, taking a large dose of 80 mg febuxostat daily can significantly reduce the uric acid level and inflammatory response, improve vascular endothelial function, enhance antioxidant ability, and improve the clinical symptoms of patients without increasing the adverse reactions to medication.

## Introduction

With the improvement of China's economy, people's living standards and lifestyles have changed greatly, the dietary structure has changed as well. As the most common metabolic-related disease, the incidence rate of hyperuricemia has gradually increased^1^ in the Asian population (5%-20%)^2^. The increase in the uric acid level results in the occurrence of gout-related clinical symptoms. As a chronic metabolic-related disease, the increased level of blood uric acid is the main pathophysiological change of hyperuricemia^3^.

Clinically, the primary principle of hyperuricemia treatment is to reduce blood uric acid levels. Commonly used uric acid lowering drugs include uricosuric agents and inhibitors of uric acid synthesis^4^. The former represented by benzbromarone, is not suitable for people with kidney damage, while the latter allopurinol, leads to exfoliative dermatitis and thus limits its clinical application^5^. Febuxostat is one of the drugs used to inhibit uric acid synthesis clinically in recent years. It can treat patients with mild to moderate renal damage and has a high long-term safety^6^.

Previously, many studies have been conducted with the shuffled quantity of doses. No unified standard for the dosage of febuxostat in the treatment of hyperuricemia in previous studies, therefore, the present study was novel, which explores the effects of febuxostat in different doses on uric acid, inflammatory factors of serum and knee articular cavity, endothelin-1 and oxidative stress in patients with gout.

## Materials and methods

### General information

Finally, 80 patients/individuals with hyperuricemia were admitted to the hospital from January 2018 to March 2020 were selected by the determination of the population size, and the diagnosis was confirmed by detection of blood biochemical uric acid and clinical manifestations. A questionnaire was designed for inclusion and exclusion criteria. All those individuals who did not fulfill the inclusion criteria were excluded from the present study. Inclusion criteria: Holmes criteria were used to diagnose the patients, patients aged 25-65 years old, with uric acid level moe than 8.0 mg/dl followed by Lee et al.^23^, and normal mental status were recruited and the consent form was signed before enrollment and the approval by the hospital ethics committee, and was obtained with the clinical trial number “ChiCTR19000282321”. Age ranges and appropriate variables are used to identify the demographic of the people which can potentially help in gaining valuable details during an analysis of their feedback to reveal if there is a strong correlation between age and disease.

All participants were treated for hyperuricemia in strict accordance with treatment guidelines, such as restriction of hyper-purine diet, strengthening exercise, recording daily water intake, adjusting work and rest time, and prohibiting smoking and drinking. These inclusion and exclusion criteria were followed by (Dalbeth et al., 2017)^21^.

For the treatment of elevated purine levels, the control group orally took febuxostat tablets (JIANGSU HENGRUI MEDICINE CO., LTD., NMPA Approval No. H20130081, batch number 201712056), 40 mg once a day, and the treatment group orally took febuxostat tablets (JIANGSU HENGRUI MEDICINE CO., LTD., NMPA Approval No. H20130081, batch number 201712056) 80 mg once a day. 4 weeks was considered as a course of treatment in both groups. The morning (7-9 AM) and evening (7-9 PM) times were prescribed for tablet intake. The idea of the timeframe was followed by a previously published study (Xu et al., 2015)^22^. Approximately 50% of the patients do not take medicine accordingly, as prescribed. But herein, in this study, most of the patients were compliant with medication.

### Treatment group indexes

**Figure 1:**
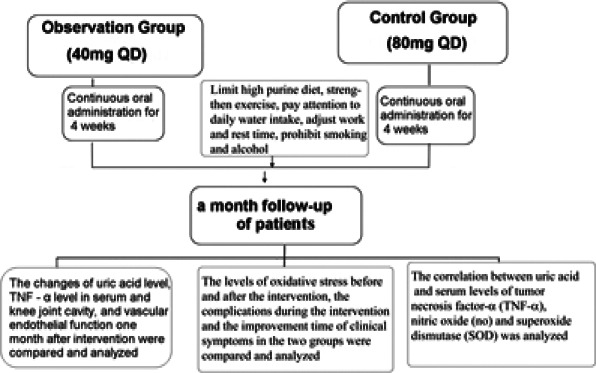
The specific experimental process

Uric acid level, inflammatory factor in serum, and knee articular cavity TNF-α of the two groups during the intervention, vascular endothelial function and regeneration ability at 1 month after the intervention, oxidative stress before and after intervention, complications during the intervention, and time to the improvement of clinical symptoms between the two groups were compared. The correlation between uric acid and serum tumor necrosis factor-α (TNF-α) level, nitric oxide (NO), and superoxide dismutase (SOD) levels were analyzed given in Figure 1.

### Evaluation Criteria

Uric acid levels were measured by Uric Acid Test Kit Sigma-Aldrich (MAK077), with normal values ranging from 208 mol/L to 428 mol/L in males and 155 mol/L to 357 mol/L in females; Tumor necrosis factor-α (TNF-α, ELISA, 5 ηg/L ~ 100 ηg/L) was used as the standard inflammatory factor in serum and knee articular cavity, vascular endothelial function indexes are mainly ET-1, ELISA, 43.5 ηg/L ~ 58.4 ηg/L), nitric oxide (NO, ELISA, the normal value of adults 13.8 µmol/L ~ 34.6 µmol/L). The indices of vascular regeneration ability are mainly vascular endothelial growth factor (VEGF, ELISA, 55.0 ηg/L ~ 90.0 ηg/L) and basic fibroblast growth factor (bFGF, ELISA, 36.9 ηg/L ~ 58.8 ηg/L). Levels of oxidative stress indexes include: malondialdehyde (MDA, 3.52 mmol/L ~ 4.78 mmol/L) and superoxide dismutase (SOD, 0.242 U/L ~ 0.620 U/L).

### Statistical analysis

All the data were normalized before the statistical analysis using the Shapiro Wilk test. All data were statistically analyzed and presented as the mean ± SD () using SPSS statistical software version 20.0. Student t-test was used to analyze statistical differences between two groups. Moreover, the data were tested by One-Way ANOVA followed by post-hoc Duncan using GraphPad Prism version 7.0. The p < 0.05 was considered statistically significant. The χ2 was used for the comparison of rates.

## Results

Previous irregular use of non-buxostat treatment, use of benzbromarone, allopurinol, colchicine, and other anti-uric acid drugs within 1 week before enrollment; have a mental illness, obvious cardiopulmonary insufficiency, severe kidney-related diseases, combined with blood system-related diseases, infections in other parts of the body, allergic to the proposed drugs. The subjects were divided into two groups according to the random number table, with 40 cases in each group. Treatment group: 31 males and 9 females, aged 25 ~ 64 years, (38.1 ± 2.6) years on average, disease course of 3 ~ 15 years, (5.6 ± 1.1) years on average, urine protein quantitative level at enrollment of (1.5 ± 0.1) g/day, 14 smokers, 12 drinkers, 25 patients with hypertension, 21 patients with type 2 diabetes, 15 patients with coronary heart disease, 20 patients with chronic obstructive pulmonary disease; Control group: 30 males and 10 females, aged 26 ~ 65 years, (38.0 ± 2.5) years on average, disease course of 3 ~ 15 years, (5.5 ± 1.0) years on average, urine protein quantitative level at enrollment of (1.6 ± 0.2) g/day, 15 smokers, 13 drinkers, 26 patients with hypertension, 20 patients with type 2 diabetes, 16 patients with coronary heart disease, 19 patients with chronic obstructive pulmonary disease. There was no significant difference in gender, age, disease duration, urinary protein quantitative level, the proportion of smoking and drinking as well as combined medical diseases between the two groups at enrollment (p>0.05).

## Methods

### Uric acid level comparisons

**Figure 2:**
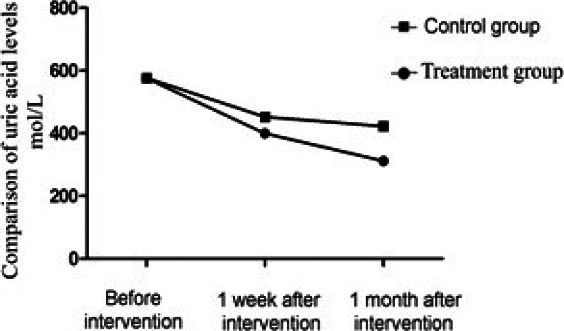
Comparison of uric acid levels between the two groups There were significant differences in uric acid between the two groups one week and one month after intervention (P < 0.05)

In the current study, Table 1 and Figure 2, raveled that there was no significant difference in uric acid levels between the two groups before intervention. In 1^st^ week and 1st month after the intervention, the uric acid level in the treatment group was significantly different and lower than that in the control group (p<0.05).

**Table 1:** Comparison of uric acid levels between the two groups during the intervention (mol/L,)

	Before intervention	1 week after intervention	1 month after intervention
Observation group	596.8±15.8	405.6±11.1	311.9±5.8
Control group	597.0±15.8	453.3±10.0	423.3±9.1
t	0.057	20.193	65.290
P	0.843	0.000***	0.000***

*** Strongly significant

### Comparison of TNF-α levels in serum and knee articular cavity between the two groups

**Figure 3:**
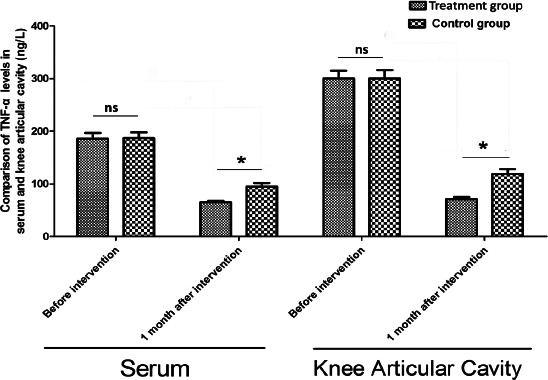
Comparison of serum and intra-articular inflammatory factor TNF - α levels between the two group @The results showed that there was no significant difference in the levels of TNF-α in serum and knee joint cavity between the two groups before the intervention (P > 0.05), * indicating that the level of TNF-α in serum and knee joint cavity of the two groups after the intervention was low, and the difference was significant (P < 0.05)

Table 2 and Figure 3 revealed that there was no significant difference in TNF-α levels of serum and knee articular cavity between the two groups (Control vs treatment) before the intervention (p>0.05). After the intervention, the TNF-α levels of serum and knee articular cavity in the treatment group were profoundly lower than in the control group (p<0.05), and TNF-α levels in serum and knee articular cavity of the two groups were significantly lower than before (p<0.05).

**Table 2:** Comparison of TNF-α levels in serum and knee articular cavity between the two groups (ng/L,)

		Serum	Knee Articular Cavity
Observation group	Before intervention	186.5±11.1	286.6±28.9
	1 month after intervention	65.6±2.8	71.5±3.9
Control group	Before intervention	186.6±11.0	286.7±29.0
	1 month after intervention	95.8±6.9	118.9±8.8
t_1_	-	66.794	46.650
P_1_	-	0.000	0.000
t_2_	-	44.226	35.018
P_2_	-	0.000	0.000
t_3_	-	0.040	0.015
P_3_	-	0.968	0.988
t_4_	-	25.650	31.145
P_4_	-	0.000	0.000

Note: t1 and P1 represent comparisons before and after intervention in the observation group, t2 and P2 represent comparisons before and after intervention in the control group, t3 and P3 represent comparisons between control group and observation group before intervention, and t4 and P4 represent comparisons between control group and observation group after intervention.

### Comparison of vascular endothelial function and regeneration ability at 1 month after intervention between the two groups

**Figure 4:**
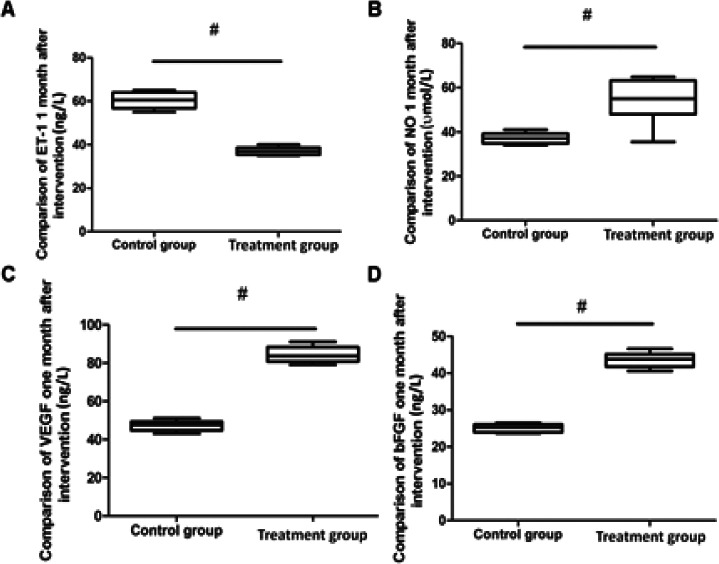
Comparison of vascular endothelial function and regeneration ability between the two groups one month after intervention A: Comparison of ET-1 One month after the intervention; B: Comparison of NO one month after the intervention; C: Comparison of VEGF one month after the intervention; D: Comparison of bFGF one month after the intervention; # P < 0.05

As given in Table 3 and Figure 4, 1 month after the intervention, the vascular endothelial cell function index NO in the treatment group was significantly higher than that in the control group (p<0.05), and the ET-1 level was significantly lower than the control group, and the levels of vascular regeneration ability indexes VEGF and bFGF were significantly higher as compared to the control group (p<0.05).

**Table 3:** Comparison of vascular endothelial function and regeneration ability at 1 month after intervention between the two groups ()

	ET-1 (ng/L)	NO (µmol/L)	VEGF (ng/L)	bFGF (ng/L)
Observation group	37.6±2.5	56.3±8.5	85.5±6.0	43.6±3.0
Control group	60.1±5.3	37.8±3.7	47.1±3.8	25.1±1.4
t	24.284	12.621	34.196	35.342
P	0.000***	0.000***	0.000***	0.000***

*** Strongly significant

### Comparison of oxidative stress between the two groups before and after intervention

There was no significant difference between MDA and SOD levels in the two groups (Control vs treatment) before the intervention (p>0.05) as shown in Table 4. After the intervention, MDA of the treatment group was significantly lower than the control group (p<0.05), while SOD was statistically higher than the control group (p<0.05), and MDA of the two groups was significantly lower than before the intervention, while SOD was higher than before (p<0.05).

**Table 4:** Comparison of oxidative stress between the two groups before and after intervention

		MDA (mmol/L)	SOD(U/L)
Observation group	Before intervention	6.5±0.8	0.3±0.1
	1 month after intervention	3.1±0.2	1.1±0.2
Control group	Before intervention	6.5±0.9	0.3±0.1
	1 month after intervention	4.9±0.4	0.6±0.2
t_1_	-	26.077	22.627
P_1_	-	0.000	0.000
t_2_	-	10.275	8.485
P_2_	-	0.000	0.000
t_3_	-	0.000	0.000
P_3_	-	1.000	1.000
t_4_	-	25.456	11.180
P_4_	-	0.000	0.000

Note: t1 and P1 represent comparisons before and after intervention in the observation group, t2 and P2 represent comparisons before and after intervention in the control group, t3 and P3 represent comparisons between control group and observation group before intervention, and t4 and P4 represent comparisons between control group and observation group after intervention.

### Comparison of complications between the two groups during interventions

As shown in Table 5, there was no statistically significant difference in the proportion of abdominal pain and diarrhea, liver damage, kidney damage, acute gout, and pruritus (p>0.05).

**Table 5:** Comparison of complications between the two groups during interventions (cases)

	Abdominal pain and diarrhea	Liver damage	Kidney damage	Acute gout	Pruritus
Observation group	1	1	2	1	1
Control group	3	5	4	3	5
χ^2^	0.263	1.622	0.180	0.263	1.622
P	0.843	0.203	0.843	0.608	0.203

### Comparison of clinical symptom improvement time between the two groups

This is a comparative study, in which movement disorder, pain, and swelling were correlated in the control group and treatment group. As shown in Table 6, the duration of movement disorder, pain, and swelling in the treatment group were significantly shorter than those in the control group (p<0.05).

**Table 6:** Comparison of clinical symptom improvement time between the two groups (d,)

	Movement disorder	Pain	Swelling
Observation group	3.1±0.2	4.6±0.5	5.3±0.6
Control group	5.3±0.3	6.8±1.1	8.5±1.6
t	38.591	11.515	11.844
P	0.000***	0.000***	0.000***

*** Strongly significant

### Correlation analysis of uric acid and serum TNF-α, NO, and SOD levels

**Figure 5:**
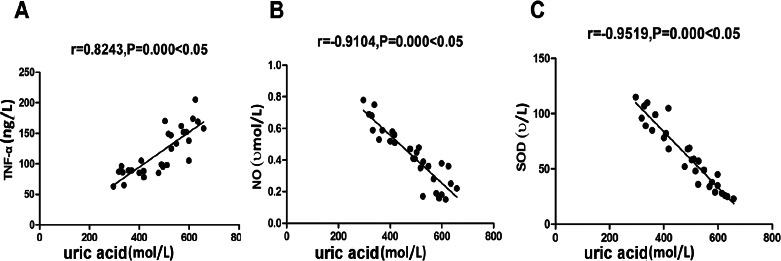
Correlation analysis A: Correlation analysis of uric acid and serum TNF-α level, B: Correlation analysis of uric acid and NO level, C: Correlation analysis of uric acid and SOD level

As shown in Figure 5, uric acid is correlated with serum TNF-α levels (p<0.05), and negatively correlated with NO and SOD levels (p<0.05).

## Discussion

With the acceleration of population aging in China, and changes in people's living habits and eating habits, the incidence of hyperuricemia has significantly increased, and the disease has the trend of rejuvenation^7^. Gout is the most prevalent inflammatory arthritis and affects about 2.5% of the general population^26^. The current epidemiology of gout indicates a rising prevalence worldwide, not only in western countries but in Asian countries as well^27^. The main cause of gout caused by hyperuricemia is the occurrence of metabolic diseases due to impaired purine metabolism^8^. Studies have shown that hyperuricemia serves as the pathophysiological basis of gout^9^. If the serum uric acid level is not effectively controlled, patients will suffer from joint destruction, liver and kidney damage, and even cardiovascular and cerebrovascular diseases as the disease progresses, which have a significant negative impact on patients' quality of life^10^. For patients with gout, allopurinol was applied by inhibiting the production of uric acid, but its long-term use displayed many adverse reactions and the patient's poor tolerance has limited its clinical application^11^. Although benzbromarone can effectively promote the excretion of uric acid, it is not suitable for people with impaired renal function^12^. Febuxostat is a new type of xanthine oxidase inhibitor with fast onset, fewer adverse reactions, and high patient compliance. Although it has been widely used in clinical practice, there is no uniform standard for clinical dosage.

For gout patients with hyperuricemia, the treatment group administered a high dose of 80 mg daily, compared with the control group of a low dose (40 mg), uric acid levels between the two groups during the intervention were contrasted. It was found that 1 week and 1 month after the intervention, the uric acid level in the treatment group was significantly lower than that of the control group, suggesting that for gout patients with hyperuricemia, daily treatment with a high dose of 80 mg can quickly and effectively reduce uric acid level. Furthermore, TNF-α levels in serum and knee articular cavity of the two groups were compared and it was found that after the intervention, TNF-α level was significantly lowered in the two groups, TNF-α level of the treatment group was lower than that of the control group, indicating that for gout patients with hyperuricemia, daily treatment with high-dose of 80 mg can reduce the level of inflammatory factors. Previously, a study reported that the one-month dose significantly reduced vascular oxidative stress^24^. Similarly, another published study revealed that one to two-month supplementation can profoundly reduce hyperuricemia and prevent the incidence of gout^25^. The vascular endothelial function and regeneration ability indexes of the two groups 1 month after the intervention were compared, and it was found that vascular endothelial cell function index NO in the treatment group was significantly higher than in the control group, and the ET-1 level was significantly lower than in the control group, the levels of vascular regeneration ability index VEGF and bFGF were significantly higher than the control group. It is suggested that for gout patients with hyperuricemia, daily treatment with a high dose of 80 mg can improve the level of vascular endothelial cells in a short time and promote the regeneration of vascular endothelial cells.

Meanwhile, the oxidative stress of the two groups before and after the intervention was compared. After the intervention, MDA of the two groups was decreased while SOD was increased, and MDA of the treatment group was lower than in the control group, and SOD was higher than the control group, suggesting that for gout patients with hyperuricemia, daily treatment with a high dose of 80 mg is of great significance to improve antioxidant ability. Moreover, in the present study, the complications during the intervention between the two groups were compared, there found no significant difference in the proportion of abdominal pain and diarrhea, liver and kidney damage, acute gout, and pruritus in the treatment group, suggesting for gout patients with hyperuricemia, daily treatment with a high dose of 80 mg do not increase the incidence of complications (p>0.05). Statistics on the improvement time of clinical symptoms between the two groups revealed the activity disorder, pain and swelling duration in the treatment group were significantly lower than the control group. It reveals that or gout patients with hyperuricemia, daily treatment with a high dose of 80 mg can significantly relieve patients' clinical symptoms and improve their life quality. Moreover, the correlation between uric acid and serum TNF-α levels, NO, and SOD levels were analyzed, and it was found that uric acid was positively correlated with serum TNF-α levels (p<0.05) and negatively correlated with NO and SOD levels. A study in 2016 reported and confirmed that there is a strong correlation between uric acid and serum TNF-α levels^30^. This further indicates that daily treatment with a high dose of 80 mg for gout patients with hyperuricemia is important to improve inflammation conditions, the activity of vascular endothelal cells, and the antioxidant ability after reducing the uric acid level. Previously, a study reported that oxidation supplementations have significantly decreased oxidative stress and disease in rheumatoid patients aged 49-60 28.

In gout patients associated with hyperuricemia, the application of febuxostat can inhibit both reduced and oxidized xanthine oxidase^13^, but mainly the fubuxostat is used to treat gout and hyperuricemia^29^, thereby promoting the formation of stable enzymes^14^, without affecting the purine metabolic pathway^15^, and does not affect the intermediate enzymes of metabolic pathways of genetic material, such as purine and pyrimidine, which can effectively reduce uric acid and incidence of adverse reactions^16^. Treating gout caused by hyperuricemia with a large dose of febuxostat (80 mg/day) can effectively and stably lower the uric acid level, reduce the incidence of acute gout, relieve patients' clinical symptoms, and increase the treatment compliance rate^17^, and high-dose febuxostat can also reduce blood creatinine and urea nitrogen levels to varying degrees, which has a certain value for improving renal function^18^. Studies have shown that high-dose febuxostat (80 mg/day) can reduce the levels of serum uric acid and sICAM-1^19^, which has a positive effect on decreasing the body's inflammatory response and promoting vascular regeneration^20^. A recent study in 2019 reported that Allopurinol and febuxostat are the primary for the treatment of gout and arthritis^27^.

## Conclusion

For hyperuricemia-induced gout patients, daily treatment with high-dose febuxostat of 80 mg can significantly reduce the level of uric acid and the body's inflammatory response, improve vascular endothelial function, and antioxidant ability, and does not increase the adverse effects of medication while improving the patient's early clinical symptoms.
